# Urinary Calculi

**Published:** 1879-09

**Authors:** 


Urinary Calculi.—On the 25th of March, 1870, Mr. S. and wife came to my office with their child, aged six years. The child, a boy, was robust and well developed. The mother stated that, during the preceding night and the forenoon of that day, the child had cried incessantly, and seemed in great agony; that during the suffering she had conjectured various things to be the matter with it, and had dosed and treated it accordingly, but all to no purpose. Finally she noticed the child strain as though endeavoring to urinate, but there was no flow of water. After sometime she discovered something hard and firm in the child's penis, just in front of the scrotum. As soon as the discovery was made she bundled up the child and started for my office. A few minutes before reaching me the child had suddenly ceased crying, and in another minute was asleep. I had the mother loosen the diaper; and immediately at the end of the penis lay a light porous gravel, of no inconsiderable size. I took it to be of the phosphatic variety. It was egg-shaped, measuring two by three lines and weighed one and a half grains.

About a week afterwards Mr. S. called at my office and left me a second gravel of the same character as the first, but somewhat larger and more rounded in form. It weighed one and a half grains, and was passed after paroxysms of great suffering.

I saw nothing of the child until July 24, when, after still greater suffering than it had yet experienced, a third gravel was passed to the meatus urinarius, where it lodged, plugging up the opening so completely that not a drop of water could be forced by. I was compelled to enlarge the opening before the gravel could be removed. It was the largest and roughest of the three, weighing three grains. I have known gravels of much smaller size cause no little pain and annoyance in an adult, and it is simply wonderful that the urethra of a child only ten months old could have passed it at all. The child was accustomed to taking no other food than milk from the mother's breast.—*Nashville Jour. Med. and Surg.*

